# *MoodyTunes:* a single cohort study of a music-based smartphone app for mental health and mood regulation in young people

**DOI:** 10.3389/fpsyg.2025.1568958

**Published:** 2025-07-29

**Authors:** Sandra Garrido, Zareen O'Keeffe, Anthony Chmiel, Katherine Boydell, Barbara Doran, Quang Vinh Nguyen

**Affiliations:** ^1^MARCS Institute for Brain, Behaviour and Development, Western Sydney University, Penrith, NSW, Australia; ^2^Sydney Conservatorium of Music, University of Sydney, Sydney, NSW, Australia; ^3^Black Dog Institute, Sydney, NSW, Australia; ^4^Faculty of Medicine, University of New South Wales, Sydney, NSW, Australia; ^5^Faculty of Transdisciplinary Innovation, University of Technology, Sydney, NSW, Australia

**Keywords:** youth mental health, digital mental health, smartphone applications, music psychology, music medicine

## Abstract

Mental health applications (apps) are proliferating to meet the needs of the increasing numbers of young people experiencing mental health challenges. However, many mental health apps for young people are either not evidence-based or fail to engage the interest of those who are not already receiving professional help. Since music listening is an activity that many young people are drawn to when experiencing high levels of psychological distress, *MoodyTunes* was developed to engage young people in learning about mental health within the context of their daily music listening activities. In this single cohort study, 70 participants aged 13–25 used *MoodyTunes* over a 4-week period. Pre- and post-intervention measures assessed mental health literacy, coping self-efficacy, depression, anxiety, and stress levels. Results demonstrated a significant increase in mental health literacy and decreases in anxiety and stress. Depression was found to have decreased, although not at a statistically significant level. No significant change in coping self-efficacy was found. These findings suggest that *MoodyTunes* may be an effective tool for improving mood regulation and psychological wellbeing in young people. Future research with larger, randomized samples and a comparative control group is recommended.

## 1 Introduction

One in seven young people experience a mental health disorder globally, with depression and anxiety among the leading causes of illness and disability among adolescents (World Health Organization, 2024). Some countries around the world have seen steep increases in the rates of mental health issues in young people in recent years. In Australia, 39% of 16–24-year-olds had a mental health disorder in 2021, up from 26% in 2007 (Australian Bureau of Statistics, 2022). Similarly, in the U.K., one in five young people aged 8–25 had a probable mental disorder compared to a rate of about one in eight in 2017 (Baker and Kirk-Wade, 2024). The U.S. has seen similar increases (Lebrun-Harris et al., 2022).

Despite high levels of need, young people typically have low rates of help-seeking behavior, and research indicates that this has not increased along with rates of mental health disorders (Brennan et al., 2021; Upton et al., 2021). Smartphone apps for supporting mental health, however, are increasingly in demand (Ariz et al., 2022). The global mental health market is projected to reach a value of $USD 20.92 billion by the year 2033, a compound annual growth rate of 16% between now and then (Straits Research Private Limited, 2024).

Although there are a wide range of mobile mental health apps, few apps have been specifically developed for promoting emotion regulation (Eisenstadt et al., 2021). Furthermore, significant concerns exist around the safety and effectiveness of such apps. Many utilize approaches that are not evidence-based (Koh et al., 2022). Others apps fail to be engaging to users—especially those targeting young people—who tend to have high expectations of the technology they use (Garrido et al., 2019b; Borghouts et al., 2021). A recent review demonstrated that many apps for young people are not designed with them, do not solve issues they care most about, fail to respect privacy and are laden with privacy concerns (Torous et al., 2018). Studies suggest that smartphone apps that increase engagement such as through use of gamification or music (Garrido et al., 2019b), and that empower the user to take care of their own mental health rather than being directive (Gotz et al., 2022; Garrido et al., 2022) are of greater appeal to young users.

Music is also a promising tool for supporting mental health in young people. Formal music therapy, a research-informed, clinical practice in which clients work with a trained music therapist, is effective in reducing symptoms of depression and anxiety in adolescents (Ishak et al., 2021). However, even outside of music therapy where no music therapist is involved, music also has the potential to help improve mental health within the context of everyday music listening. Teenagers aged 13–18 years spend around 2 h per day listening to music (Dredge, 2019), and this amount can increase when young people are experiencing psychological distress (Stewart et al., 2019). Listening to music can help young people with both social wellbeing and emotion regulation (Papinczak et al., 2015). However, this reliance on music listening is not always helpful, with studies showing that many young people are attracted to music that can exacerbate negative thinking and depression (Ter Bogt et al., 2019; Larwood and Dingle, 2021; Garrido and Schubert, 2015; Powell et al., 2021). Thus, music can serve as a useful entry point into learning about mental health, providing both a tool that young people are naturally drawn to during periods of psychological distress and a unique opportunity to foster self-awareness and promote healthier coping strategies.

*MoodyTunes*^1^ is a smartphone app that was co-designed with young people and that draws on principles of cognitive behavioral therapy to help young people learn better self-management of mood in the context of their music-listening behaviors. It is designed to help young people become more aware of how their own choices influence their mood and wellbeing, using music as a medium to subtly enhance mental health literacy and promote healthy mood regulation strategies through experiential learning. As users listen to music on *Spotify* as part of their usual daily activities, the *MoodyTunes* app prompts users to record how specific music affects their mood. It creates “feel better” playlists to challenge habitual listening patterns and foster greater self-awareness. Additionally, the app encourages users to reflect on thoughts and emotions triggered by music and introduces techniques such as cognitive reframing and challenging automatic negative thoughts. It also directs users to short, informative articles on various mental health topics, and helps them to understand when professional help might be needed and how to get it (see Supplementary material for screenshots of the *MoodyTunes* app).

*MoodyTunes* was developed based on extensive research into the effect of music on mood and theory from both the music psychology and music therapy literature. In the early stages of development, co-design workshops were conducted in which young people identified the strengths and limitations of existing mental health apps and proposed ideas for integrating music and wellbeing research into an app (Garrido et al., 2019a). Young people were also involved in the creation of a mock-up and prototype, concept testing, a think-aloud study (Duguid et al., 2022) and further iterations of design and development (Garrido et al., 2022, 2024). The current study aims to conduct a preliminary investigation into the effectiveness of *MoodyTunes* in increasing mental health literacy and coping self-efficacy, as well as reducing symptoms of depression, anxiety, and stress. The study will add to the body of knowledge about how music can be used to enhance mental health and wellbeing in young people while demonstrating the potential of the *MoodyTunes* app to contribute to this.

It was hypothesized that:

H1: Users will report increased mental health literacy and coping self-efficacy after a 4-week period in which they used *MoodyTunes*.
H2: Users will report decreased depression, stress and anxiety after a 4-week period in which they used *MoodyTunes*.

## 2 Methods and materials

### 2.1 Study design

The study employed a single-group pretest-posttest design to evaluate initial trends on the primary outcome measures among young people who use *MoodyTunes* for 4 weeks. Given the pilot nature of the study and the early-stage development of the app, a randomized controlled trial was not considered appropriate at this stage. Participant engagement with the app was also assessed to explore the acceptability and appeal of the app to users. Potential differences in age and gender in response to the app were also explored.

### 2.2 Participants

Purposive sampling was used to recruit Australian youth, with an attempt made to balance recruitment across genders and two age groups: those aged 13–18 years and those aged 19–25. Participants were recruited from among undergraduate students at an Australian University through internal emails and noticeboards, via social media advertising to the general public and snowballing. Recruitment emails and social media read: “Do you like music? Would you like to know more about how you can use music to manage your moods? Researchers from Western Sydney University would like you to help us test and evaluate a smartphone app designed to help young people learn how to use music to effectively manage their moods. You get to download the app for free and use it for 4 weeks and will receive a $100 gift voucher upon completion.”

Ninety-two participants were initially recruited to take part in the study. Of these, 70 participants produced complete data at both baseline and follow-up. The responses for the remaining 22 participants were excluded due to incomplete data, representing a completion rate of 76%. There were no significant differences in age group, gender, or baseline DASS scores between completers and non-completers. Participants were eligible to participate if they were aged between 13 and 25 years of age and had access to a smartphone on which Spotify could be installed. Participants were not required to use a paid version of Spotify since the app works both with free and premium versions of Spotify. Participants were excluded if they had used *MoodyTunes* before or did not meet the inclusion criteria. Participants were given a $100 gift voucher for completion.

The mean age of participants was 18.0 years (SD = 4.0), with 42 participants aged 18 or younger (60%). For analysis, participant ages were grouped as either 18 and below (*n* = 42), or 19 and above (*n* = 28). This variable is hence referred to as “Age group.” The final sample of 70 participants included 22 males (31.4%), 39 females (55.7%), six participants (8.6%) who selected “Non-binary/Third gender,” and three (4.3%) who selected “Prefer not to say.” Due to the smaller sample sizes, the latter two gender categories were collapsed into a single category for all analyses (“Non-binary/Prefer not to say”).

### 2.3 Procedures

Ethics approval was obtained from the Human Research Ethics Committee of Western Sydney University (approval number H14725). Participants or their parents (if aged 15 or under) were emailed an information sheet and pre-screening questions to determine eligibility to participate. Eligible participants and the parents of those aged 15 or under signed a consent form which was returned to members of the research team by email. Once consent had been provided, participants were given a unique numerical identifier and emailed a link to the pre-intervention survey. Upon completing the pre-intervention survey, participants were emailed instructions about how to download and set up the *MoodyTunes* app on their devices. The instructions included information on connecting *MoodyTunes* to a Spotify account and how to enter their numerical identifier into the app. Participants were then asked to use *MoodyTunes* at least twice weekly for 4 weeks. A member of the research team contacted them shortly after this step to ensure that they had been able to install and use the app and then again at the 2-week mark to troubleshoot any technical issues. Once the 4-week period had ended, participants were emailed a link to the post-intervention survey.

### 2.4 Materials and measures

*MoodyTunes* is available for both Android and Apple devices, and participants used their own personal devices to access the app. Pre and post-study surveys were completed on Qualtrics. In the pre-study survey participants first completed 5-items relating to demographics and music listening behaviors, including the average amount of time they spend listening to music and the devices and platforms they typically use for music listening. They then completed several baseline measures (listed below), which were also completed in the follow-up post-study survey.

Participants were asked both at baseline and at post-study to rate their agreement with an additional three items specifically related to understanding the relationship between music and mental health: (i) Music is one of those things that always helps people when they are feeling depressed or anxious, (ii) Sometimes music can make someone feel worse if they are feeling depressed, and (iii) I feel confident in choosing music that can help me manage my moods. Participants indicated agreement or disagreement with the items using a Likert scale of 1 (Strongly Disagree) to 5 (Strongly Agree). The first question, being a statement that is incorrect, was negatively scored to indicate an accurate understanding of the relationship between music and mental health. These items were summed to create a total Music and Mental Health (MusicMH) score. These three items returned a reliability score of Cronbach's α = 0.61 at baseline, and 0.60 at post-study suggesting an acceptable level of reliability. Additionally, in the post-study survey participants were asked which type of Spotify account they had used in conjunction with *MoodyTunes* (a premium, free, or family account), and completed a series of questions examining responses to the app drawn from the Mobile Application Rating Scale (MARS; Stoyanov et al., 2015). Participants also provided general feedback in response to an open-ended question, which read: “Do you have any other comments you would like to make about the app concept or design or anything else relevant?”

The Coping Self-Efficacy Scale (CSES; Chesney et al., 2006) was used to measure confidence in coping behaviors pre- and post-survey when faced with stressful life challenges. The CSES is a 26-item scale in which participants report how confident they are in performing adaptive coping behaviors (e.g., “keep yourself from feeling lonely” and “Take your mind off unpleasant thoughts”) by responding to an 11-point Likert Scale (0 = “cannot do at all” and 10 = “certain can do”). The CSES exhibits commendable reliability, with Chesney et al. (2006) reporting a Cronbach's alpha of 0.95. Cronbach's alpha for the current study was α = 0.95 at baseline and α = 0.96 at post-study.

The Mental Health Literacy Scale (MHLS; O'Connor and Casey, 2015) and the Mental Health Literacy Questionnaire—Young Adult (MHLq-ya; Dias et al., 2018) were used to measure participants' understanding of mental health. The MHLS is a 35-item scale which assesses six aspects of mental health literacy. Since *MoodyTunes* primarily aims to reduce stigma and promote help-seeking only 20 items relating to these aspects of mental health were retained in the current study. The wording of some items was modified slightly to be more relevant to Australian youths. The MHLS exhibits good reliability, with O'Connor and Casey (2015) reporting a Cronbach's alpha coefficient of α = 0.87. Cronbach's alpha for the current study was α = 0.85 at baseline and α = 0.91 at post-study. The MHLq-ya is a 29-item Scale used to assess mental health experience, knowledge, attitudes, and mental health-seeking behaviors in young people using a 4-point Likert scale. Some items were omitted for irrelevancy to the current study, with a modified version of 22 items being used here. The original MHLq-ya exhibits good reliability, with Dias et al. (2018) reporting a Cronbach's alpha coefficient of α = 0.84. Our study returned a reliability score of α = 0.56 at baseline and α = 0.58 at post-study, suggesting this to be a less reliable scale than the MHLS in the current sample.

The Depression and Anxiety Scale (DASS-21; Lovibund and Lovibund, 1995) was used to measure Stress, Depression and Anxiety pre and post-study. The DASS-21 is a 21-item scale where participants indicated how much a statement applied to them (e.g., “I felt that I had nothing to look forward to” or “I found it hard to wind down”) using a 4-point Likert Scale of 0 (“Never”) to 3 (“Almost Always”). The DASS-21 exhibits excellent reliability, with Lovibund and Lovibund (1995) reporting internal consistency of Depression α = 0.90; Anxiety α = 0.84; Stress α = 0.90. Patrick et al. (2010) found an overall internal consistency of α = 0.92 when assessing validity for children and adolescents aged 11 to 17 years. Cronbach's alphas for the current study were Depression 0.93 at baseline and 0.91 at post-study; Anxiety 0.87 at baseline and 0.87 at post-study; Stress 0.87 at baseline and 0.88 at post-study.

A shortened version of the MARS (Stoyanov et al., 2015) was used post-survey to rate participants' overall experience using *MoodyTunes*. The MARS is a 23-item rating scale used to assess mobile health apps' engagement, quality, aesthetics, and impact. The present study used a 16-item scale, with some questions omitted or modified to be more relevant to *MoodyTunes*. Participants rated their experience using a 5-point Likert scale with varying wording for response categories depending on the wording of the question. Higher numbers indicated more positive responses to the app (see Table 1). For example, Item 1 asked “Was *MoodyTunes* interesting to use?” with responses ranging from 1 (“Not interesting at all”) to 5 (“Extremely interesting”), and Item 3 asked “How easy was it to learn to use this app?” with responses ranging from 1 (“Confusing, complicated”) to 5 (“Able to use immediately, intuitive, simple”) (see Table 1). One item (Item 9) was rated on a 3-point Likert scale. Subscale totals were calculated for engagement, aesthetics, quality and impact, with a single item assessing user perceptions of functionality. The MARS exhibits good reliability, with Stoyanov et al. (2015) reporting a Cronbach's alpha score of 0.85. The Cronbach's alpha for the current study was α = 0.90.

**Table 1:** Descriptive statistics for app ratings based on Mobile Application Rating Scales (MARS).

**Scale**	***M***	***SD***	**Min**.	**Max**.
1. Was *MoodyTunes* interesting to use?	3.3	0.9	1	5
2. Do you think the content of *MoodyTunes* is appropriate for your age group?	4.1	0.8	1	5
3. How easy was it to learn to use this app?	3.7	1.1	1	5
4. What do you think of the design of the screens in this app?	4.0	0.9	2	5
5. What do you think of the graphics used for icons/buttons?	4.0	0.7	2	5
6. How good does the app look?	4.2	0.7	3	5
7. How likely would you be to recommend *MoodyTunes* to others?	3.7	0.9	2	5
8. How many times do you think you would use the app in the next 12 months?	3.4	1.2	1	5
9. Would you pay for this app?	1.5	0.6	1	3
10. What is your star rating for this app?	3.6	0.7	2	5
11. This app helped me become more aware of the importance of addressing mental health problems	3.5	1.0	1	5
12. This app helped me to better understand how music can influence mental health	4.1	0.9	1	5
13. This app helped change my attitudes toward mental health	3.2	1.1	1	5
14. This app helped to increase my motivation to do things that can benefit my mental health	3.5	1.0	1	5
15. This app increased my confidence in my ability to manage my moods and mental health	3.6	1.1	1	5
16. This app encouraged me to get professional help	2.8	1.1	1	5
MARS Engagement	3.7	0.7	1	5
MARS Aesthetics	4.1	0.5	2	5
MARS Quality	3.1	0.8	1	5
MARS Impact	3.4	0.8	1	5

Min. and Max. refer to the minimum and maximum response recorded for that scale. For all scales N = 70. All items were measured on 5-point scales, apart from Item 9 which was measured on a 3-point scale.

### 2.5 Data analysis

Descriptive statistics were generated to assess general acceptability and ratings of user experience of the app on the MARS. Repeated measures ANOVAs were performed with scores on outcome measures over Time (pre-study, post-study) as the dependent variable, Age Group and Gender as the independent variables. Data for the three DASS subscales were not normally distributed, and so Wilcoxon non-parametric tests were used to assess changes over time on these outcome measures. Analyses are based on median rather than mean scores for these three tests only.

## 3 Results

### 3.1 General response to the app

The majority of participants reported listening to music at least 1 h per day or more (1–2 h: *n* = 20, 28.6%; 2–5 h: *n* = 22, 31.4%; 5–8 h: *n* = 10, 14.3%; 8 h+: *n* = 2, 2.9%). The most commonly used devices for listening to music were iPhones (*n* = 56, 80%) and computers (*n* = 33, 47.1%). Most participants reported that for their general music listening (i.e., external to this study) they used Spotify for listening to music (*n* = 65, 92.9%), primarily a premium account (*n* = 34, 48.6%). YouTube was the next most frequently used listening platform (*n* = 27, 38.6%).

Descriptive statistics for individual items and the four MARS sub-scales are listed in Table 1. Ratings for all items were above the mid-point, indicating a generally positive response to the app. The highest rated aspect was related to app aesthetics (Item 6; MARS Aesthetics), with the lowest ranking being for item 16 (“This app encouraged me to get professional help”). However, it was notable that 15 participants agreed (*n* = 10, 14.1%) or strongly agreed (*n* = 5, 7.0%) that using the app had encouraged them to get professional help. On average, the four MARS subscales returned a mean of 3.6 (SD = 1.1).

Feedback in response to the open-ended question provided some useful suggestions for future improvement of the app. In particular, some participants wanted to be able to rate songs more easily upon opening the app rather than waiting for notifications (*n* = 7). A number of other participants reported some functionality issues that had impacted their overall experience with the app (*n* = 11). However, comments were generally positive, and participants expressed their enjoyment of the app and belief that it could make a difference to mental health: “I have nothing to add, this app was honestly so interesting and amazing to use. I'm so honored I could be a part of using this app” (16 year old, male).

### 3.2 Mental health literacy

**Figure 1:**
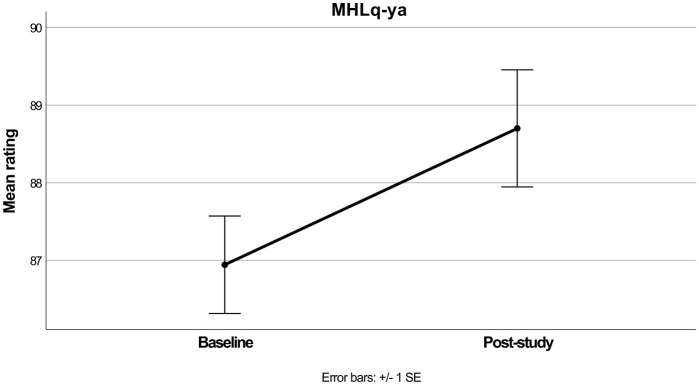
Mean MHLq-ya scores before and after the intervention.

The repeated measures ANOVA for MHLq-ya was significant [*F*_(1, 69)_ = 6.51, *p* = 0.013, $\eta_{p}^{2}$ = 0.086], with mean ratings increasing from baseline to post-study as shown in Table 2 and Figure 1. A subsequent repeated measure ANOVA was performed, adding both Age group and Gender as separate independent variables. The main effect (time) remained significant [*F*_(1, 64)_ = 4.89, *p* = 0.031, $\eta_{p}^{2}$ = 0.071], although there were no significant interactions with Age Group [*F*_(1, 64)_ = 1.02, *p* = 0.315, $\eta_{p}^{2}$ = 0.016] or Gender [*F*_(1, 64)_ = 0.27, *p* = 0.762, $\eta_{p}^{2}$ = 0.008].

**Table 2:** Descriptive statistics for outcome variables at baseline and post-study.

**Independent variable**	**Baseline**			**Post-study**		
	***M***	***SD***	***Median***	***M***	***SD***	***Median***
CSE	169.0	42.1	–	169.1	54.1	–
DASS anxiety	15.7	6.2	14.0	14.6	6.0	13.5
DASS depression	15.4	6.4	14.0	14.9	6.2	12.5
DASS stress	18.1	5.7	17.5	16.7	5.8	16.0
MHLq-ya	86.9	5.2	–	88.7	6.3	–
MH literacy	173.2	11.6	–	177.5	13.9	–
Music MH	9.5	1.6	–	10.1	1.5	–

Median values are additionally reported for the three DASS-21 subscales, as these were atypically distributed scales and so needed to be examined via Wilcoxon rank-based nonparametric tests. All other tests were examined by ANOVA, using mean ratings.

**Figure 2:**
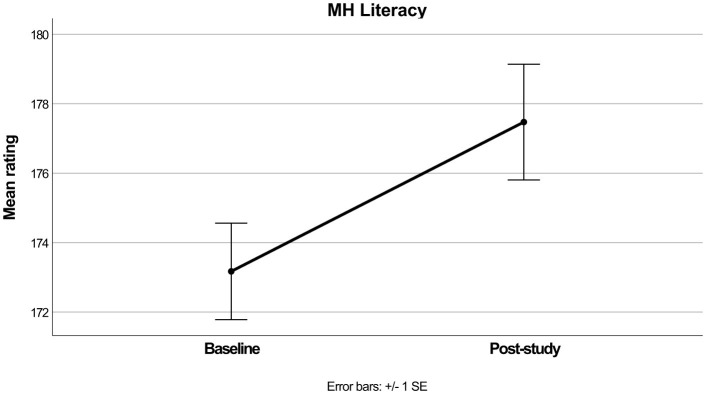
Mean MH literacy scores before and after intervention.

The repeated measures ANOVA for MH Literacy also produced a significant main effect [*F*_(1, 69)_ = 12.39, *p* < 0.001, $\eta_{p}^{2}$ = 0.152], in which mean ratings increased over time (see Table 2 and Figure 2). When Age group and Gender were added as interacting variables the main effect again remained significant [*F*_(1, 64)_ = 8.62, *p* = 0.005, $\eta_{p}^{2}$ = 0.119] although as above the interactions with Age Group [*F*_(1, 64)_ = 3.03, *p* = 0.087, $\eta_{p}^{2}$ = 0.045] and Gender were non-significant [*F*_(1, 64)_ = 0.15, *p* = 0.857, $\eta_{p}^{2}$ = 0.005].

**Figure 3:**
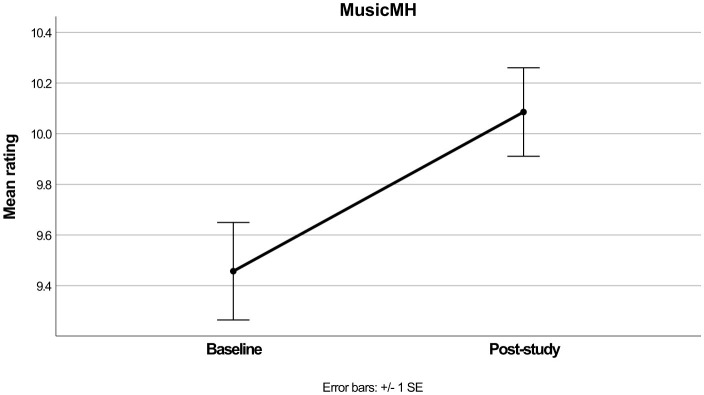
Mean MusicMH scores before and after intervention.

The repeated measures ANOVA for MusicMH produced a significant main effect [*F*_(1, 69)_ = 13.04, *p* < 0.001, $\eta_{p}^{2}$ = 0.159], and as per Table 2 and Figure 3 the mean values increased from baseline to post-study. Again, while the main effect remained significant [*F*_(1, 64)_ = 4.60, *p* = 0.036, $\eta_{p}^{2}$ = 0.067] the interactions with Age group [*F*_(1, 64)_ = 0.75, *p* = 0.390, $\eta_{p}^{2}$ = 0.012], and Gender [*F*_(1, 64)_ = 1.72, *p* = 0.188, $\eta_{p}^{2}$ = 0.051] did not reach significance.

### 3.3 Coping self-efficacy

The repeated measures ANOVA for CSE did not reach significance [*F*_(1, 69)_ = 0.01, *p* = 0.973, $\eta_{p}^{2}$ < 0.001], and in the follow-up ANOVA neither did the main effect (*p* = 0.598) or the interactions by Age group (*p* = 0.518) and Gender (*p* = 0.343). As shown in Table 2, there was little change in CSE ratings over time.

### 3.4 Depression, anxiety, and stress

Young people in this study demonstrated extremely severe levels of Depression, Anxiety, and Stress at baseline (Table 2). Wilcoxon tests for both DASS Anxiety (*z* = −2.53, *p* = 0.011) and DASS Stress (*z* = −2.62, *p* = 0.009) showed significant decreases in Median values over time, whereas the decreasing values for DASS Depression only approached significance (*z* = −1.79, *p* =0.074). Distributions for all three DASS tests are plotted in the Supplementary Section 2 (Supplementary Figures 1–3).

## 4 Discussion

This study was a preliminary investigation exploring whether a cohort of young people would experience changes in mental health literacy, coping self-efficacy, depression, anxiety and stress after using the *MoodyTunes* app over a 4-week period. Results indicated that young people did experience increases in mental health literacy as well as decreases in stress and anxiety, after 4 weeks of app usage. General responses to the app were also positive, with rating scales indicating that young people found the app aesthetically pleasing, somewhat engaging and of good quality. They also believed that the app increased their awareness and confidence in managing mental health challenges, although there were no significant changes in coping self-efficacy over time. User engagement ratings on the MARS and changes in symptoms over time are comparable to that of other apps at similar stages of development (Serlachius et al., 2021; Elledge et al., 2023).

Helping young people to increase their mental health literacy is no small accomplishment. Previous research has shown that young people with high levels of mental health literacy are less likely to experience psychological distress than other youths, a relationship that is mediated by increased psychological resilience (Zhang et al., 2023). They are also more likely to seek professional help when needed, which can improve overall mental health outcomes (Bennett et al., 2023).

Nevertheless, challenges exist to engaging young people in learning more about mental health. Indeed, young people often prefer the anonymity, ease of access and non-threatening nature of digital mental health interventions, even if they acknowledge that face-to-face support might be better (Pretorius et al., 2019; Garrett et al., 2024). Despite lower ratings for the questionnaire item about seeking professional help (Item 16) than for other items, it is notable that a number of young people who participated in the study reported having been encouraged to seek professional help after using *MoodyTunes*. This study has therefore demonstrated that *MoodyTunes* holds potential to provide an engaging way for young people to become more aware of mental health challenges and how to manage them. This may be particularly valuable for those who may experience disparities in health services or who may not get professional help for other reasons (Amos et al., 2023).

The results of this study contribute to the existing body of evidence supporting the potential benefits of digital mental health interventions based on cognitive behavioral therapy for adolescents (Csirmaz et al., 2023). Familiarizing users with mood reflection techniques as used in CBT can help improve mental health outcomes (Barcak et al., 2022), and music can be an effective way to do this (McFerran et al., 2018). Furthermore, empirical research has consistently demonstrated that listening to music can be effective in regulating mood in young people, suggesting its usefulness as a means for developing healthy mood regulation strategies (Dingle and Fay, 2017). Young people in this study also tended to have increased confidence and understanding of how music can influence mental health by the end of the study.

Nevertheless, despite improvement in stress and anxiety in the current sample, it is important to note that these were non-clinical outcome measures and those participants were not necessarily representative of clinical populations despite their high levels of depression, anxiety, and stress at baseline. Therefore, these findings do not demonstrate the capacity for *MoodyTunes* to reduce symptoms of significant mental illnesses. Rather, the reduction in stress and anxiety in participants found in this study suggest the public health benefits of *MoodyTunes* as a tool for increasing self-management of non-clinical levels of mood disturbances. Furthermore, the lack of a control group and the fact that participants were only required to use the app a minimum of two times per week suggest that caution should be used in attributing these changes solely to use of *MoodyTune*s. Further research using a control group and randomization will be needed to confirm a causal relationship.

The high baseline DASS scores in our sample suggest that participants were experiencing substantial psychological distress at the time of recruitment. This may reflect a self-selection bias, where participants may have been drawn to the study due to its mental health focus. This suggests that caution should be taken in generalizing the findings to all young people, particularly those not experiencing high levels of distress. Future studies should explore the effectiveness of *MoodyTunes* in more diverse samples, including those with varying levels of baseline distress.

The finding in this study that age group and gender did not have an influence on outcomes is encouraging. Mental health issues can escalate in severity from ages 11 to 14 (Patalay and Fitzsimons, 2018), a group which tends to be particularly underserved by mental health services (National Mental Health Commission, 2023). Young males can also be especially difficult to engage in learning about mental health and tend to have poorer rates of mental health literacy than females in the same age group (Rice et al., 2018). The current study demonstrates that *MoodyTunes* may be a suitable tool for both younger age groups and young males. Indeed, a previous evaluation of *MoodyTunes* found that the app was particularly appealing to young people aged 17 and under Duguid et al. (2022).

## 5 Conclusion

The observed positive changes in mental health literacy and mood experienced after using *MoodyTunes* for a period of 4 weeks is notable given the increasing reports of young people experiencing mental health challenges. Feedback from participants indicates that further improvements to the app could be made to enhance functionality and appeal. However, these preliminary findings underscore the potential of this digital mental health intervention as a discreet and accessible tool to address the underutilization of mental health support among youth and to further encourage help-seeking behavior, overall suggesting that *MoodyTunes* is suitable for further investigation in a controlled trial.

## Data Availability

The datasets presented in this article are not readily available because extended consent to use the data in studies for a similar purpose was obtained from participants. Under these conditions data is not publicly available, but can be shared with researchers for use in similar studies. Requests to access the dataset should be directed to SG: s.garrido@westernsydney.edu.au.

## Footnotes

^1^ https://www.moodytunes.com.au
