# Supplementary material for: MoodyTunes: a single cohort study of a music-based smartphone app for mental health and mood regulation in young people
Source: Front Psychol. 2025 Jul 29;16:1568958. doi: 10.3389/fpsyg.2025.1568958 (PMC12341477; doi:10.3389/fpsyg.2025.1568958)
Supplement: Supplementary file 1 [file Supplementary_file_1.pdf]

Supplementary Material

MoodyTunes: A single cohort study of a music-based smartphone app for mental health and mood regulation in young people

Sandra Garrido, Zareen O'Keeffe, Anthony Chmiel, Katherine Boydell, Barbara Doran, Quang Vinh Nguyen

1 MoodyTunes app screenshots

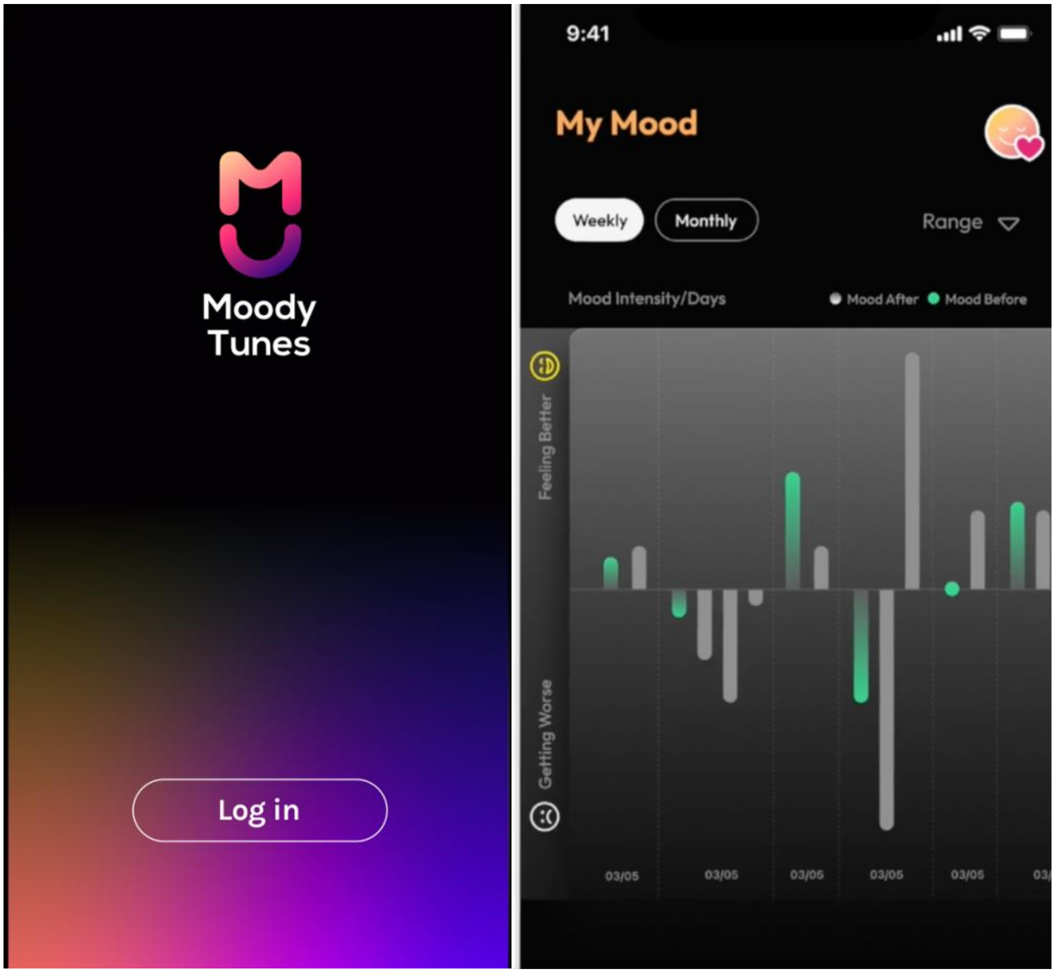

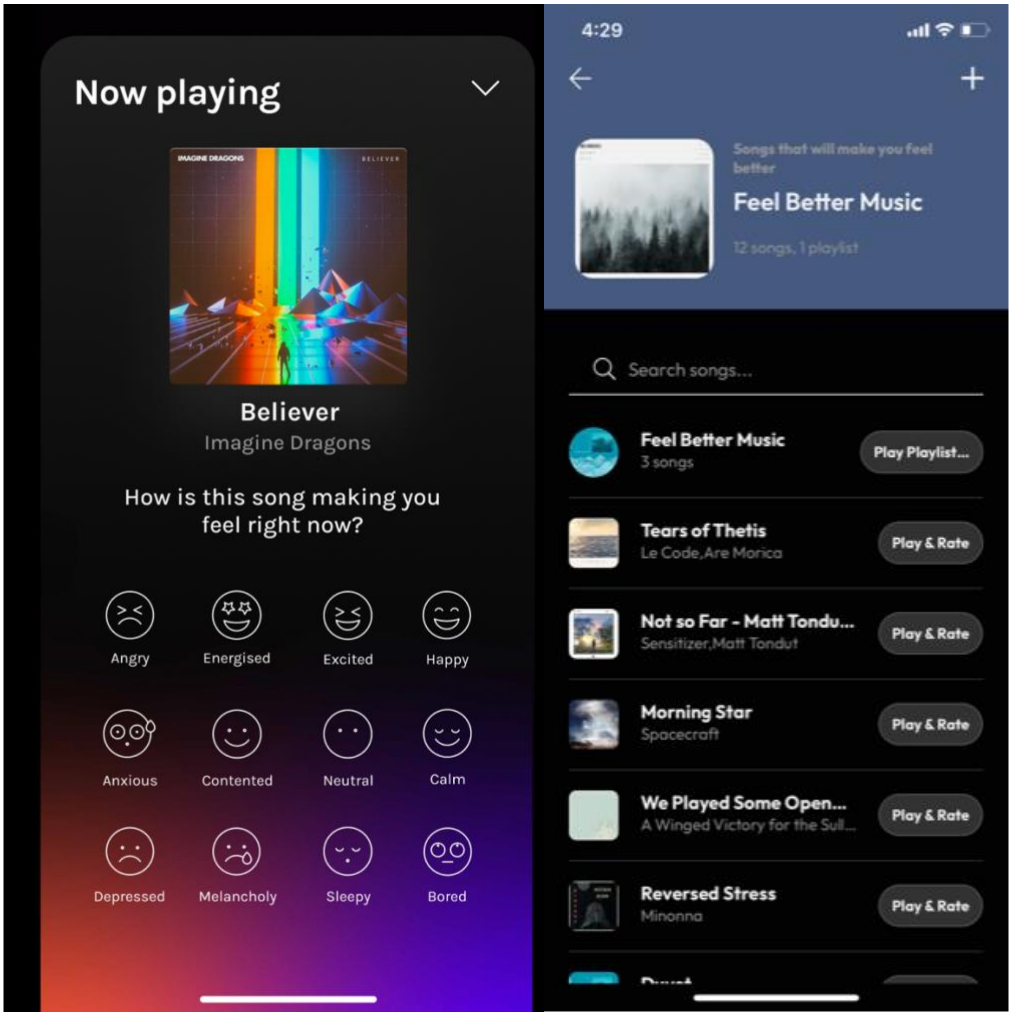

Now playing
Believer
Imagine Dragons
How is this song making you feel right now?
Angry
Energised
Excited
Happy
Anxious
Contented
Neutral
Calm
Depressed
Melancholy
Sleepy
Bored
4:29
Songs that will make you feel better
Feel Better Music
12 songs, 1 playlist
Search songs...
Feel Better Music
3 songs
Play Playlist...
Tears of Thetis
Le Code,Are Morica
Play & Rate
Not so Far - Matt Tondu...
Sensitizer,Matt Tondut
Play & Rate
Morning Star
Spacecraft
Play & Rate
We Played Some Open...
A Winged Victory for the Sul...
Play & Rate
Reversed Stress
Minonna
Play & Rate

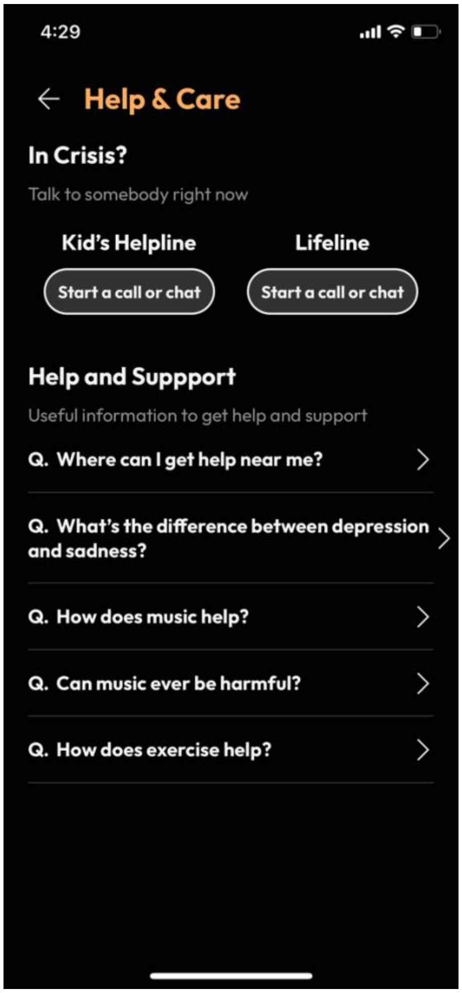
4:29
Help & Care
In Crisis?
Talk to somebody right now
Kid's Helpline
Start a call or chat
Lifeline
Start a call or chat
Help and Suppport
Useful information to get help and support
Q. Where can I get help near me?
Q. What's the difference between depression and sadness?
Q. How does music help?
Q. Can music ever be harmful?
Q. How does exercise help?

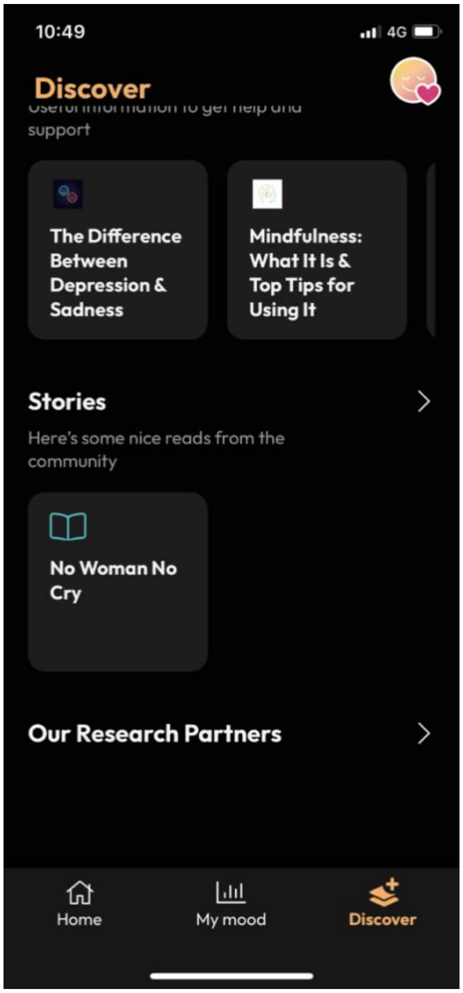
10:49
4G
Discover
support
The Difference Between Depression & Sadness
Mindfulness: What It Is & Top Tips for Using It
Stories
Here's some nice reads from the community
No Woman No Cry
Our Research Partners
Home
My mood
Discover

2 Wilcoxon non-parametric test plots

Figures are provided here for Wilcoxon tests on the three DASS-21 subscales, to better illustrate the spread of data. The depicted data are based on median values, as is typical for Wilcoxon rank-based tests.

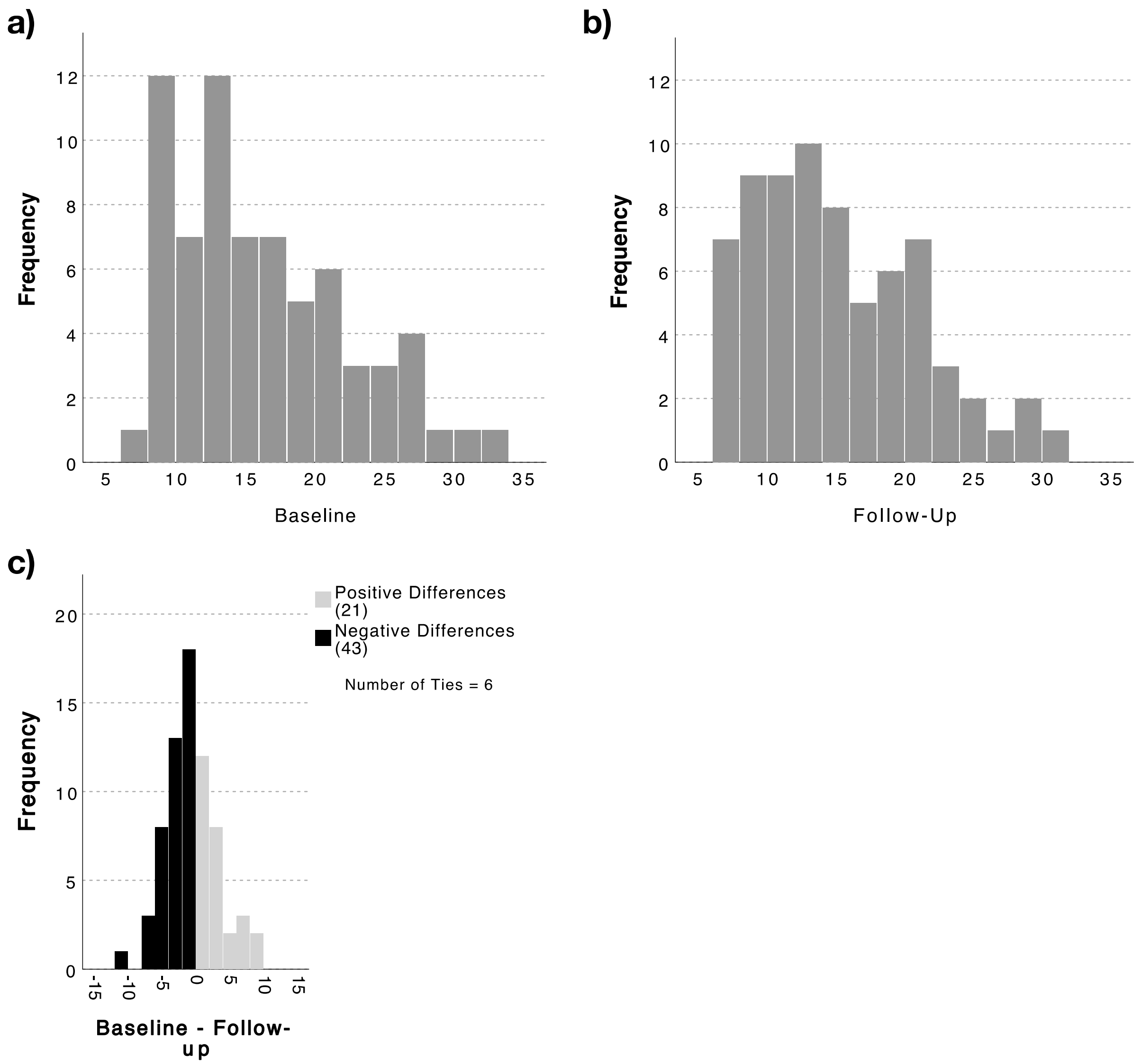

Supplementary Figure 1.
Panels 1a to 1c depict the Wilcoxon test examining the DASS Anxiety scale at baseline and post-study sessions. As per the main text, this test was significant (p = .011), and showed a decrease in Median values over time. 1a) shows DASS Anxiety at Baseline, 1b) at the Post-study session, and 1c) shows the positive versus negative movement between those two time points.

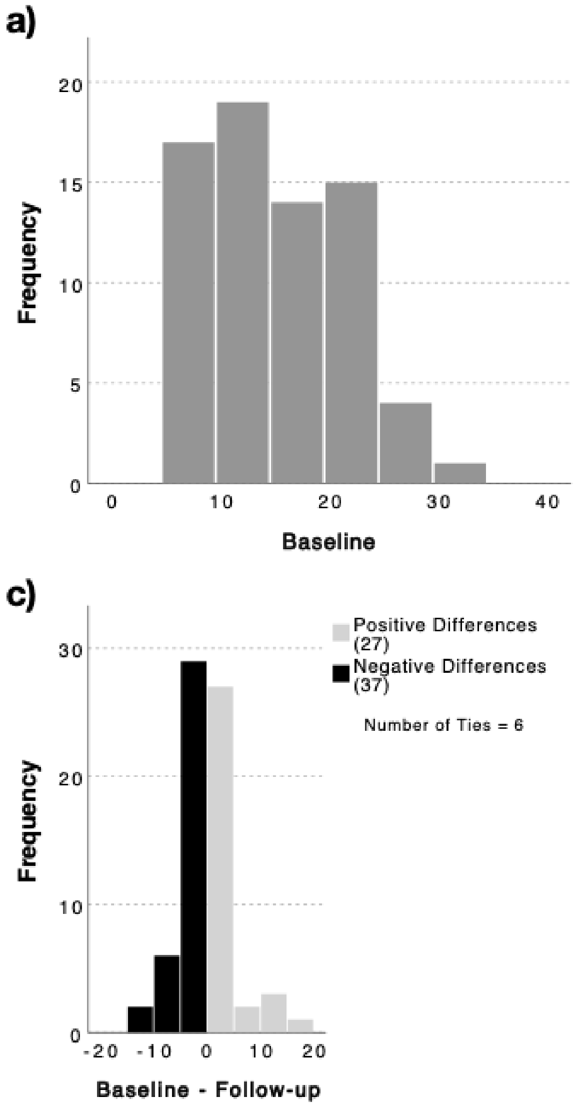

Supplementary Figure 2.
Panels 2a to 2c depict the Wilcoxon test examining the DASS Depression scale at baseline and post-study sessions. As per the main text, this test approached significance (*p* = .074). Median values show a decrease over time. 2a) shows DASS Depression at Baseline, 2b) at the Post-study session, and 2c) shows the positive versus negative movement between those two time points.

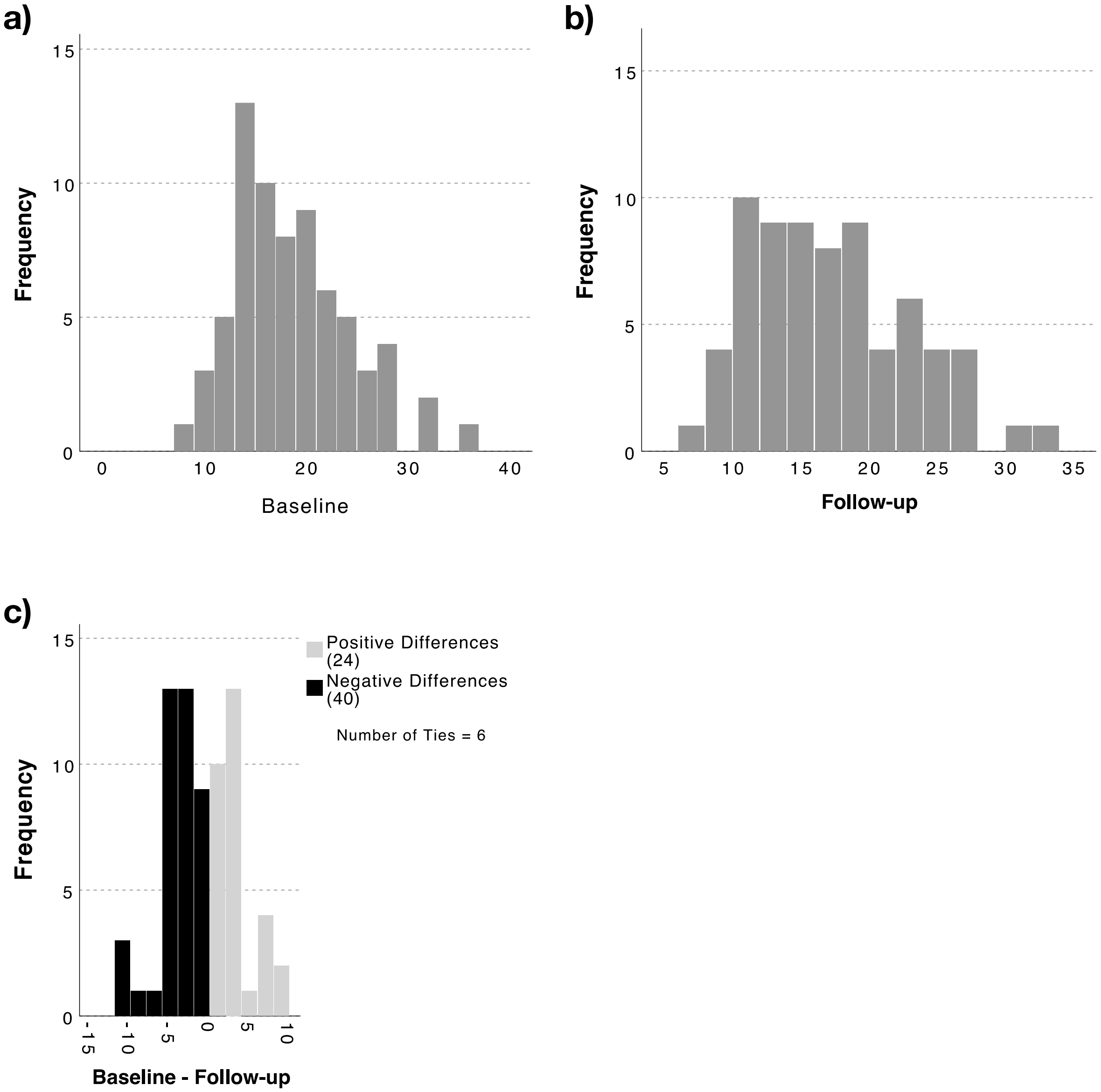

Supplementary Figure 3.
Panels 3a to 3c depict the Wilcoxon test examining the DASS Stress scale at baseline and post-study sessions. As per the main text, this test was significant (p = .009), and showed a decrease in Median values over time. 1a) shows DASS Stress at Baseline, 1b) at the Post-study session, and 1c) shows the positive versus negative movement between those two time points.
